# Integrating Large Language Models Into UAE Community Pharmacies: Pharmacists′ Perspectives on Benefits, Concerns, and Implementation Barriers

**DOI:** 10.1155/ijta/8034289

**Published:** 2026-01-14

**Authors:** Anan S. Jarab, Ahmad Z. Al Meslamani, Walid Al-Qerem, Huda Alyafeai, Yazid N. Al Hamarneh, Judith Eberhardt

**Affiliations:** ^1^ Department of Clinical Pharmacy, Faculty of Pharmacy, Jordan University of Science and Technology, Irbid, Jordan, just.edu.jo; ^2^ College of Pharmacy, Al Ain University, Abu Dhabi, UAE, aau.ac.ae; ^3^ Faculty of Pharmacy, Al-Zaytoonah University of Jordan, Amman, Jordan, zuj.edu.jo; ^4^ College of Pharmacy, Fatima College of Health Sciences, Abu Dhabi, UAE, fchs.ac.ae; ^5^ Department of Pharmacology, Faculty of Medicine and Dentistry, University of Alberta, Edmonton, Canada, ualberta.ca; ^6^ School of Social Sciences Humanities and Law, Teesside University, Middlesbrough, UK, tees.ac.uk

**Keywords:** barriers and concerns, community pharmacy, digital health integration, healthcare technology adoption, large language models, pharmacists′ perspectives

## Abstract

**Background:**

The UAE′s rapid economic growth and adoption of advanced healthcare technologies necessitate understanding pharmacists′ perspectives on large language models (LLMs) to address implementation challenges and align with the nation′s digital health initiatives.

**Aim:**

This study explored UAE pharmacists′ perceived benefits, concerns, and barriers to LLM adoption, as well as factors contributing to heightened concerns in community pharmacies.

**Methods:**

A survey‐based cross‐sectional study was conducted among 528 community pharmacists (51.3% female) in the UAE between October and November 2024. Pharmacists completed a validated questionnaire assessing socio‐demographic information, perceived benefits, concerns, and barriers related to LLM use. Binary logistic regression was applied to identify factors associated with concerns about LLMs.

**Results:**

The least‐perceived benefits of LLMs included providing around‐the‐clock support (37.3%), designing personalized care plans (74.4%), and improving patient outcomes (77.0%). Barriers included the need for human supervision (54.7%), insufficient training (32.4%), lack of pharmacy‐focused LLM programs (28.4%), and inadequate resources (28.4%). Key concerns were technical failures or downtime (97.5%), hacking vulnerabilities (97.2%) and limited capacity for empathy, cultural understanding, or ethical considerations in healthcare (95.6%). Increased age was significantly associated with greater concerns (OR = 1.124, p < 0.001). Conversely, pharmacists with master′s or doctoral degrees (OR = 0.483, p = 0.008) and those likely to use LLMs in the future (OR = 0.357, *p* < 0.001) expressed fewer concerns.

**Conclusion:**

The integration of LLMs into community pharmacy practice faces challenges, including hacking risks, security vulnerabilities, insufficient empathy, and technical failures. Targeted interventions such as enhanced training, robust security measures, and tailored LLM solutions are essential to address these barriers and support safe adoption in pharmacy settings.

## 1. Introduction

Pharmacy practice is an ever‐evolving field that has transitioned from a primarily dispensing‐focused role to a comprehensive, patient‐centered clinical profession. As part of this ongoing transformation, advanced technologies, specifically large language models (LLMs), are increasingly being integrated to drive healthcare innovation [1]. LLMs represent a subset of natural language processing (NLP), a branch of artificial intelligence (AI), and are designed to understand, produce, and modify text on a scale that closely resembles human language [2]. A notable example of an LLM is OpenAI′s ChatGPT, which was introduced in November 2022 and now has over 200 million weekly active users [3].

By enabling activities including knowledge management, data analysis, process automation, and system integration, LLMs can improve pharmacy operations, potentially streamline supply chain procedures, and enhance clinical decision‐making [4]. However, their implementation raises significant concerns, including the difficulty of maintaining models up to date given the high retraining costs and potential performance degradation; the pivotal role and limitations of prompt quality (stemming from context and token constraints); considerable privacy and security vulnerabilities (e.g., data leakage, prompt injection, and unauthorized code execution); and the risk of AI hallucinations, where models may confidently generate misinformation [4].

In the United Arab Emirates (UAE), rapid economic growth has led to important technological advancements in pharmacy practice, including electronic health records, telepharmacy, robotics, and AI tools [5–7]. Although the UAE has embraced several digital health projects, little is known about how community pharmacists perceive advanced AI tools like LLMs, specifically regarding their benefits, implementation difficulties, and ethical considerations. A qualitative study found that pharmacists in the UAE viewed ChatGPT as a tool to improve medication compliance, utilization, management, safety, and adherence [8]. Nevertheless, because ChatGPT relies on outdated databases or limited information, pharmacists believed it might provide incomplete or erroneous suggestions. Therefore, the present study was aimed at assessing pharmacists′ perceived benefits, concerns, and barriers related to integrating LLMs in UAE community pharmacies. Understanding these factors is essential to addressing potential challenges and optimizing their adoption in a way that aligns with the UAE′s rapidly advancing healthcare infrastructure and commitment to digital innovation.

## 2. Materials and Methods

### 2.1. Study Design and Participants

The present survey–based cross‐sectional study targeted community pharmacists in the UAE who completed a self‐administered validated questionnaire between October and November 2024. Pharmacists eligible for participation were those who had graduated from universities accredited by the UAE Ministry of Higher Education and were registered as community pharmacists with the UAE Ministry of Health. Pharmacists with less than 6 months of work experience and pharmacy technicians were excluded. To facilitate data collection, each research pharmacist visited community pharmacies in their respective residential areas and distributed the survey link via Google Forms to eligible participants. To minimize the risk of selection bias from targeting only specific groups of pharmacists, the survey link was disseminated widely to reach a broad audience. Each research pharmacist was based in a different geographic region, allowing for wider coverage across both urban and rural settings. They were instructed to approach a diverse mix of pharmacy types, including independent and chain pharmacies, to ensure representation across various practice environments. The survey was conducted anonymously to encourage honest responses, further reducing selection bias. Pharmacists who agreed to participate were required to provide informed consent. On average, participants spent approximately 10 min completing the questionnaire.

### 2.2. Ethics Statement

The current research received ethical approval from the research ethics committee at Al Ain University–Abu Dhabi Campus (Ref #: COP/AREC/AD/03).

### 2.3. Study Instrument

The study questionnaire was developed based on a review of relevant, previously published research studies [9–19]. The survey began with a brief introduction outlining the objectives of the study and providing assurances about voluntary participation, anonymity, and confidentiality of the findings. The first part of the questionnaire evaluated sociodemographic and practice‐related information, including age, gender, educational degree attained, type of pharmacy, years of work experience, workload in the community pharmacy, and the likelihood of recommending the use of LLMs in pharmacy practice. The second part assessed the perceived benefits of using LLMs in community pharmacies on a 5‐point Likert scale ranging from “strongly disagree” to “strongly agree.” The third part evaluated perceived concerns using a yes/no response format. Benefits were assessed using a Likert scale to capture varying levels of agreement, while concerns were evaluated using yes/no responses to identify specific issues that may act as barriers in a straightforward manner. The final section explored barriers to using LLM services in community pharmacies. A panel of experts reviewed the relevancy and comprehensiveness of the study survey. In a pilot study involving 10 community pharmacists, the survey′s relevancy and question clarity were assessed. The data collected during the pilot test were excluded from the final analysis. Cronbach′s alpha of 0.77 and 0.79 demonstrated the reliability of the perceived benefits and perceived concerns scales, respectively. On average, participants took 10 min to complete the study questionnaire.

### 2.4. Sample Size Calculations

Sample size was calculated using the following equation [20]:

n=z2p 1−pd2

Here, *p* represents the predicted percentage (assumed to be 0.5 to account for maximum variability) and *d* represents the margin of error (0.05). The *Z* value corresponding to a 95% confidence level (1.96) was used in the calculation. As a result, a sample size of 385 was determined to be necessary.

### 2.5. Statistical Analysis

The Statistical Package for the Social Sciences (SPSS), Version 28, Illinois, New York, United States) was used to conduct statistical analyses. *Q*‐*Q* plots and the Kolmogorov–Smirnov test indicated that the continuous variables were not normally distributed. Categorical variables were presented as frequencies and percentages, while continuous variables were presented as median (interquartile range (IQR)). A binary logistic regression analysis was performed to identify the variables significantly associated with the outcome variable—pharmacists perceived concerns about utilizing LLMs in community pharmacies. Age, gender, educational attainment, job title, pharmacy type, years of experience, workload, and the likelihood of using LLMs in the future, were the predictor variables. *p* values below 0.05 were considered statistically significant.

## 3. Results

The total number of pharmacists who completed the questionnaire was 528, of whom 271 (51.3%) were female, 421 (79.7%) held a bachelor′s in pharmacy, and 351 (66.5%) worked in independent community pharmacies (Table 1). Participants′ median age was 31.0 years (IQR: 28.0–34.0). Among the participants, 363 (68.8%) reported that they were likely to use LLMs in the future. Younger participants (median age 31 vs. 33; *p* = 0.001) were significantly more likely to express this intention. Educational level (*p* = 0.006), years of experience (*p* < 0.001), and workload (*p* < 0.001) were significantly associated with participants′ likelihood of using LLMs in the future.

**Table 1:** Sociodemographic profile of the participants (*n* = 528).

**Item**	**Total,** **n** **(%)**	**Likely to use LLMs in the future**	**Unlikely to use LLMs in the future**	**p**
Age, median (IQR)	31.0 (28.0–34.0)	31 (28–34)	33 (29–35)	**0.001**
Sex				0.897
Female	271 (51.3%)	187 (69.0%)	84 (31.0%)	
Male	257 (48.7%)	176 (68.5%)	81 (31.5%)	
Educational level				**0.006**
Bachelor in pharmacy	421 (79.7%)	303 (72.0%)	118 (28.0%)	
Master or PhD	90 (17.0%)	51 (56.7%)	39 (43.3%)	
Doctor in pharmacy	17 (3.2%)	9 (52.9%)	8 (47.1%)	
Type of pharmacy				0.054
Independent community pharmacy	351 (66.5%)	112 (63.3%)	65 (36.7%)	
Chain community pharmacy	177 (33.5%)	251 (71.5%)	100 (28.5%)	
Years of experience				**0.001**
Less than 1 year	47 (8.9%)	38 (80.9%)	9 (19.1%)	
1–10 years	449 (85.0%)	312 (69.5%)	137 (30.5%)	
More than 10 years	32 (6.1%)	13 (40.6%)	19 (59.4%)	
Workload				**< 0.001**
Somewhat busy	174 (33.0%)	96 (55.2%)	78 (44.8%)	
Somewhat unbusy	141 (26.7%)	127 (901%)	14 (9.9%)	
Very busy	38 (7.2%)	15 (39.5%)	23 (60.5%)	
Very unbusy	2 (0.4%)	0 (0.0%)	2 (100.0%)	
Neutral	173 (32.8%)	125 (72.3%)	48 (27.7%)	

*Note:* Comparisons between categorical variables (sex, educational level, type of pharmacy, years of experience, and workload) were performed using Chi‐square tests. For age, which was not normally distributed, the Mann–Whitney *U* test was used. All tests were two‐tailed, and a *p* value of less than 0.05 was considered statistically significant. Bold indicates significant results.

Among the participants, 124 (23.5%) disagreed and 73 (13.8%) strongly disagreed that LLMs can provide support around the clock, particularly in emergency situations (Table 2). Additionally, 321 (60.8%) disagreed and 72 (13.6%) strongly disagreed that LLMs can be used to design personalized care plans for patients. Furthermore, 348 (65.9%) disagreed and 63 (11.9%) strongly disagreed that LLMs could help improve patients′ health outcomes.

**Table 2:** Pharmacists′ opinions on the benefits of LLMs in pharmacy practice.

**Items**	**Strongly agree**	**Agree**	**Neutral**	**Disagree**	**Strongly disagree**
LLMs can provide support around the clock, particularly in emergency situations	10 (1.9%)	184 (34.8%)	137 (25.9%)	124 (23.5%)	73 (13.8%)
LLMs can multitask and analyze data more quickly than humans while consistently providing optimal choices	55 (10.4%)	420 (79.5%)	39 (7.4%)	7 (1.3%)	7 (1.3%)
LLMs enhance accessibility by communicating in multiple languages, benefiting diverse patient populations and healthcare professionals	73 (13.8%)	428 (81.1%)	23 (4.4%)	4 (0.8%)	0 (0.0%)
LLMs is crucial for advancing my career as a pharmacist	5 (0.9%)	291 (55.1%)	134 (25.4%)	75 (14.2%)	23 (4.4%)
LLMs allow me to stay up to date with the latest developments in pharmacy	42 (8.0%)	446 (84.5%)	38 (7.2%)	1 (0.2%)	1 (0.2%)
LLMS can handle the routine inquires, freeing up pharmacists′ time for more complex task	52 (9.8%)	413 (78.2%)	56 (10.6%)	5 (0.9%)	2 (0.4%)
In complex or uncommon scenarios, LLMs can provide insights based on the latest medical knowledge and guidelines assisting in making informed decisions	14 (2.7%)	453 (85.8%)	47 (8.9%)	6 (1.1%)	8 (1.5%)
LLMs can contribute to reducing medical errors	77 (14.6%)	391 (74.1%)	33 (6.3%)	21 (4.0%)	6 (1.1%)
LLMs can enhance efficiency in medication dispensing and management	12 (2.3%)	448 (84.8%)	57 (10.8%)	7 (1.3%)	4 (0.8%)
LLMs can be used to design personalized care plans for patients	5 (0.9%)	101 (19.1%)	29 (5.5%)	321 (60.8%)	72 (13.6%)
LLMs provide quick responses, reducing wait times and increasing their satisfaction.	13 (2.5%)	470 (89.0%)	40 (7.6%)	5 (0.9%)	0 (0.0%)
LLMs can enhance patient engagement and education	6 (1.1%)	400 (75.8%)	63 (11.9%)	12 (2.3%)	47 (8.9%)
LLMs help improve patients′ health outcomes	3 (0.6%)	49 (9.3%)	65 (12.3%)	348 (65.9%)	63 (11.9%)

**Figure 1:**
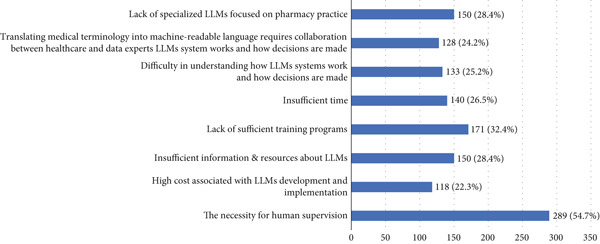
Barriers to implementing LLMs in pharmacy practice.

Participants identified several barriers to LLMs in pharmacy practice (see Figure 1). In particular, 289 (54.7%) indicated the necessity for human supervision over LLMs. Additionally, 171 (32.4%) identified a lack of sufficient training as a potential barrier. Furthermore, 150 (28.4%) of participants reported that the lack of pharmacy‐focused LLM programs and insufficient resources are barriers to using LLMs in pharmacy practice.

Overall, 293 (55.5%) expressed concern about the use of LLMs in pharmacy practice. Among the pharmacists included in this study, 515 (97.5%) believed that technical failures or downtime could disrupt pharmacy operations and patient care (Table 3). Moreover, 513 (97.2%) were concerned that LLMs are vulnerable to hacking or other security threats as a software system. Furthermore, 505 (95.6%) were concerned that LLMs have a low capacity to handle conversations requiring human empathy, an understanding of cultural contexts, or ethical considerations in healthcare.

**Table 3:** Concerns about implementing LLMs in pharmacy practice.

**Item**	**Yes**	**No**
Technical failures or downtime could disrupt pharmacy operations and patient care	515 (97.5%)	13 (2.5%)
LLMs is vulnerable to hacking or other security threats as a software system	513 (97.2%)	15 (2.8%)
LLMs interacts with users by gathering and analysing data, which can lead to privacy concerns if not managed appropriately	450 (85.2%)	78 (14.8%)
LLMs has low capacity to handle nuanced conversations that require human empathy, understanding of cultural contexts, or ethical considerations in healthcare	505 (95.6%)	23 (4.4%)
LLMs has the capability to learn biases and discriminatory patterns from the data it is trained on, potentially resulting in biased responses	45 (8.5%)	483 (91.5%)
LLMs misinterpreting medical information or providing inaccurate recommendations could lead to incomplete or inappropriate advice	309 (58.5%)	219 (41.5%)
LLMs are incapable of making critical decisions, such as those concerning end‐of‐life care, and may produce unreliable reports in such instances	251 (47.5%)	277 (52.5%)
Patients or pharmacists may not fully understand the limitations of LLMs, leading to over‐reliance or misinterpretation of its responses	420 (79.5%)	108 (20.5%)
Over‐reliance on LLMs might decrease chances for face‐to‐face interactions with pharmacists, potentially affecting the quality of patient‐pharmacist relationships	419 (79.4%)	109 (20.6%)
I am concerned that patients may distrust healthcare providers because of their reliance on LLMs	426 (80.7%)	102 (19.3%)
I am worried that LLMs might replace my role as a pharmacist	379 (71.8%)	149 (28.2%)
In general, LLMs‐related concerns exceed their benefits	409 (77.5%)	119 (22.5%)

Age was significantly positively associated with concern about LLMs (OR = 1.124, *p* < 0.001, 95% CI [1.061, 1.191]) (Table 4). Individuals with a master′s degree or PhD were less likely to be concerned compared to those with lower degrees (OR = 0.483, *p* = 0.008, 95% CI [0.282, 0.828]). Additionally, respondents who reported being likely to use LLMs in the future had lower odds of being concerned (OR = 0.357, *p* < 0.001, 95% CI [0.234, 0.547]). Gender, type of pharmacy, years of experience, and perceived workload did not show statistically significant associations with concerns about LLMs in this model.

**Table 4:** Predictors of being concerned about implementing LLMs in pharmacy practice.

**Independent variable**	**AOR**	**p**	**95% confidence interval**		**VIF**
			**Lower**	**Upper**	
Age	1.124	< 0.001	1.061	1.191	1.119
Sex					
Female (Ref)	1.000				
Male	0.944	0.765	0.646	1.379	1.160
Educational level					1.116
Bachelor in pharmacy (Ref)	1.000				
Doctor in pharmacy	0.394	0.118	0.122	1.269	
Master or PhD	0.483	0.008	0.282	0.828	
Type of pharmacy					1.048
Chain community pharmacy (Ref)	1.00				
Independent community pharmacy	0.903	0.617	0.606	1.346	
Years of experience					1.762
Less than 1 year (Ref)	1.00				
1–10 years	0.705	0.346	0.340	1.460	
More than 10 years	0.364	0.142	0.095	1.404	
Workload					1.037
Very busy	1.000				
Somewhat busy	1.231	0.582	0.587	2.583	
Neutral	0.749	0.454	0.352	1.594	
Somewhat unbusy	0.929	0.854	0.423	2.038	
Very unbusy	0.750	0.844	0.043	13.180	
How likely are you to use LLMs in your future clinical practice?					1.251
Unlikely (Ref)	1.000				
Likely	0.357	< 0.001	0.234	0.547	

## 4. Discussion

This study provides valuable insights into the perceptions of community pharmacists in the UAE about the integration of LLMs into pharmacy practice. It highlights potential benefits, such as improved clinical decision‐making and streamlined processes, as well as challenges and barriers that could hinder their widespread adoption. For policymakers, healthcare managers, and tech developers aiming to promote safe and efficient AI‐driven solutions, a thorough understanding of diverse perspectives is essential. By focusing on pharmacists′ opinions, stakeholders can develop targeted strategies, ranging from rigorous data security measures to specialized training programs, that address the challenges of implementing LLMs in a community pharmacy setting.

In the current study, most participants expressed a willingness to use LLMs in pharmacy practice. This finding is consistent with a previous study conducted in the UAE, which reported a high willingness among community pharmacists to adopt AI technologies [18]. Several factors were identified as significantly associated with this inclination, including age, educational level, years of experience, and the perceived level of busyness within their work environment. For instance, younger pharmacists were more likely to adopt LLMs in pharmacy practice, possibly due to greater familiarity with digital tools [21, 22]. Previous studies confirm the relationship between sociodemographic characteristics and the adoption of AI tools, including LLMs [23], which highlights the importance of targeted strategies to provide equitable access, education, and support for diverse pharmacist groups.

Regarding the views of pharmacists on the benefits of LLMs in pharmacy practice, our findings indicate skepticism about LLMs′ capability to provide around‐the‐clock support, design personalized care plans, or improve patient outcomes. A recent survey from Egypt indicated that 65.9% of pharmacists either disagreed or were neutral about the use of ChatGPT for evaluating patients′ medical information and delivering personalized medical advice [24]. These findings may be explained by evidence showing that LLMs often produce “hallucinations” or unsubstantiated information [25]. Consequently, pharmacists′ reluctance to entrust LLMs with vital clinical tasks is unsurprising, given the need for verified, evidence‐based guidance to safeguard patient welfare. Ongoing research into statistical techniques to detect confabulations, or random and incorrect generations, highlights the potential for future systems to identify prompts that may yield erroneous outputs.

In this study, over half of respondents believed that human oversight of pharmacy operations is essential, and nearly a third cited insufficient training resources as a key barrier. Moreover, participants identified the lack of pharmacy‐focused LLM solutions tailored for medication therapy management and patient counselling, as a potential barrier to adopting LLMs in pharmacy practice. These results are consistent with broader conversations on AI in pharmacy, which have identified related challenges [26]. For instance, no AI system can fully replace human judgement in complex clinical decision‐making, and adoption has been hindered by issues such as high installation costs, mistrust of AI, concerns about job security, and data privacy risks. Hence, a multifaceted approach addressing worker training, stakeholder involvement, appropriate resource allocation, and secure machine learning techniques is necessary for the effective integration of AI. Experts emphasize that AI should serve as a tool to support pharmacy staff rather than replace them, with the goal of streamlining processes and enhancing patient care while preserving the human elements essential to therapeutic decision‐making [26].

Our findings show that nearly half of the participating pharmacists expressed concerns about adopting LLMs in pharmacy settings. Specifically, they were concerned about security and ethical issues, including potential hacking or software vulnerabilities, and inadequate capacity for empathy or cultural sensitivity. These findings match concerns reported in prior literature. Studies from Egypt [24] and Jordan [27] reported several pharmacists′ concerns regarding the use of LLMs, including data protection and confidentiality, potential privacy breaches, cybersecurity threats, job displacement, and the lack of legal regulation. Lima et al. [28] reviewed 14 studies on the efficacy of ChatGPT in pharmacy practice; nearly all studies identified significant limitations in ChatGPT′s accuracy, dependability, and capacity to provide personalized patient care. The review found that ChatGPT tended to generate inaccurate or incomplete answers to clinical enquiries, struggled with specific prescription‐related prompts (such as drug–herb interactions), and frequently fell short of the thorough, patient‐centered perspective that a human pharmacist would provide. These findings correspond with broader concerns expressed by pharmacists, namely, ChatGPT′s propensity to produce “hallucinations,” and highlights the need for prudent, evidence‐based integration of LLMs into practice.

When testing predictors for these concerns, we found that older pharmacists are more likely to express concern about LLMs. Previous studies show significant differences in concerns about LLMs across age groups [27]. For instance, Hasan and colleagues found a negative correlation between total concern score and age [27]. A plausible explanation for this finding may be that older adults often exhibit increased resistance or skepticism towards new technology owing to perceived complexity, fear of obsolescence, or established professional routines. We also found that pharmacists who were holding a master′s degree or PhD were less likely to express concerns, potentially due to their extensive academic training, which fosters confidence in evaluating and integrating new technologies. Conversely, the literature indicates that individuals with advanced technological proficiency and AI expertise may be more cognizant of the latent drawbacks of AI, leading to heightened concerns [27]. This disparity suggests that formal education offers a theoretical perspective that balances advantages and risks, whereas practical AI experience reveals more complex systemic issues, such as data biases and ethical dilemmas, necessitating greater vigilance.

Overall, these findings highlight the urgent need for robust governance and focused approaches to integrate LLMs into pharmacy practice in the UAE. The previous literature showed that UAE pharmacists are evolving in terms of responsibilities and duties [29]. Given the significant concerns about hacking, software vulnerabilities, and the lack of empathy and cultural sensitivity in the technology [30], policymakers should prioritize developing explicit legislation addressing data security, patient confidentiality, and pharmacist oversight. To ensure that both younger and more experienced professionals can effectively use LLMs in complex clinical scenarios, health authorities and educational institutions should work together to provide extensive training programs tailored to diverse pharmacist demographics. These steps, underpinned by robust legislative frameworks and evidence‐based recommendations, may promote broader adoption of AI‐driven solutions among pharmacy professionals while preserving innovation and patient safety.

## 5. Study Limitations

It is important to consider the limitations of this study when interpreting its findings. First, the use of convenience sampling may not have represented all community pharmacists in the UAE, potentially overrepresenting subgroups who were more accessible or willing to participate, which might have led to selection bias. Second, the cross‐sectional design only captured participants′ views at a single moment in time, making it challenging to establish causation or track changes in attitudes or behaviors over time. Third, several important topics were beyond the scope of this study, such as the specific ways in which LLMs are used in pharmacies, how well they perform in clinical or operational settings, and their impact on standard pharmacy processes. Lastly, although large chain pharmacies dominate the national landscape, our sample included a higher proportion of independent pharmacists. This may reflect both regional variations and the recruitment method and therefore may not fully represent the broader distribution of pharmacy settings across the UAE and, therefore, should be interpreted with caution.

Future research utilising longitudinal or experimental methodologies and examining diverse healthcare environments, may provide an understanding of these unexplored issues, thereby offering more substantial evidence to inform policy and practice concerning LLM implementation.

## 6. Conclusion

The integration of large LLMs into pharmacy practice holds great promise, yet it is accompanied by notable challenges that require careful consideration. In this study, approximately two‐thirds of the pharmacists expressed readiness to use LLMs, with this inclination primarily influenced by factors such as age, education, years of experience, and workload. Moreover, over half of the participants identified human oversight as crucial and pointed to insufficient training and a lack of pharmacy‐specific LLM solutions as barriers to adoption. The challenges of integrating LLMs into pharmacological practice are underscored by concerns about hacking, security vulnerabilities, insufficient empathy, and potential technical failures. Age was significantly correlated with concerns about LLMs, while pharmacists with master′s or doctorate degrees were less concerned. Consequently, targeted interventions, such as enhanced training, robust security measures, and tailored LLM solutions, are essential for the effective and safe integration of LLMs into community pharmacy settings.

## Conflicts of Interest

The authors declare no conflicts of interest.

## Funding

No funding was received for this manuscript.

## Supporting information


**Supporting Information** Additional supporting information can be found online in the Supporting Information section. STROBE Checklist, outlining the reporting standards followed in this study.

## Data Availability

The data that support the findings of this study are available from the corresponding author upon reasonable request.
